# 4-Bromo-2-{(*E*)-3-[1-(hydroxy­imino)eth­yl]phenyl­imino­meth­yl}phenol

**DOI:** 10.1107/S1600536809048855

**Published:** 2009-11-21

**Authors:** Li Xu, Lei Wu

**Affiliations:** a School of Chemical and Biological Engineering, Lanzhou Jiaotong University, Lanzhou 730070, People’s Republic of China

## Abstract

In the title compound, C_15_H_13_BrN_2_O_2_, he oxime unit adopts an *E* conformation with respect to the O—H group. A classical intra­molecular O—H⋯N hydrogen bond results in the formation of a six-membered ring. The crystal structure is stabilized by inter­molecular O—H⋯N hydrogen bonds between the hydr­oxy groups and the oxime N atoms. In addition, the crystal structure also features short inter­molecular Br⋯Br short contacts with a distance of 3.8768 (5) Å.

## Related literature

For background to Schiff bases, see: Dong *et al.* (2007 ▶, 2008 ▶); Wang *et al.* 2009 ▶). For background to oximes, see: Golovnia *et al.* (2009 ▶); Liu *et al.* (2008 ▶); Dong *et al.* (2009*a* ▶); Öztürk *et al.* (2009 ▶). For the synthesis, see: Dong *et al.* (2009*b* ▶).
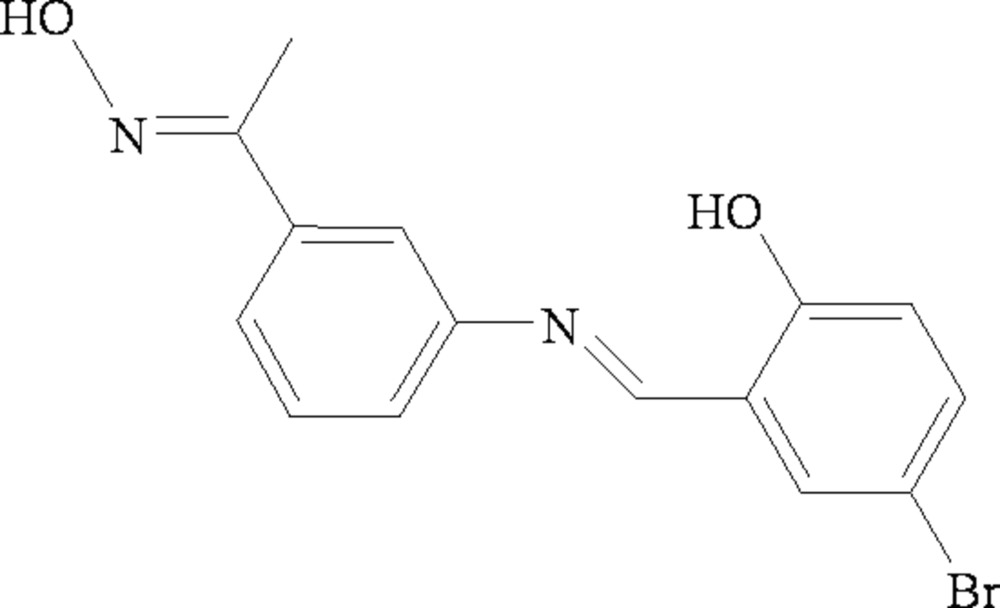


## Experimental

### 

#### Crystal data

C_15_H_13_BrN_2_O_2_
*M* *_r_* = 333.18
Monoclinic, 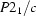

*a* = 17.020 (2) Å
*b* = 6.1676 (7) Å
*c* = 13.693 (1) Å
β = 96.461 (1)°
*V* = 1428.3 (3) Å^3^
*Z* = 4
Mo *K*α radiation
μ = 2.88 mm^−1^
*T* = 298 K
0.45 × 0.20 × 0.10 mm

#### Data collection

Siemens SMART 1000 CCD area-detector diffractometer
Absorption correction: multi-scan (*SADABS*; Sheldrick, 1996 ▶) *T*_min_ = 0.357, *T*_max_ = 0.762
6906 measured reflections
2492 independent reflections
1799 reflections with *I* > 2σ(*I*)
*R*_int_ = 0.051

#### Refinement

*R*[*F* ^2^ > 2σ(*F* ^2^)] = 0.039
*wR*(*F* ^2^) = 0.100
*S* = 0.99
2492 reflections
182 parameters
H-atom parameters constrained
Δρ_max_ = 0.47 e Å^−3^
Δρ_min_ = −0.32 e Å^−3^

### 

Data collection: *SMART* (Siemens, 1996 ▶); cell refinement: *SAINT* (Siemens, 1996 ▶); data reduction: *SAINT*; program(s) used to solve structure: *SHELXS97* (Sheldrick, 2008 ▶); program(s) used to refine structure: *SHELXL97* (Sheldrick, 2008 ▶); molecular graphics: *SHELXTL* (Sheldrick, 2008 ▶) and *DIAMOND* (Brandenburg, 1998 ▶); software used to prepare material for publication: *SHELXTL*.

## Supplementary Material

Crystal structure: contains datablocks global, I. DOI: 10.1107/S1600536809048855/lx2124sup1.cif

Structure factors: contains datablocks I. DOI: 10.1107/S1600536809048855/lx2124Isup2.hkl

Additional supplementary materials:  crystallographic information; 3D view; checkCIF report

## Figures and Tables

**Table 1:** Hydrogen-bond geometry (Å, °)

*D*—H⋯*A*	*D*—H	H⋯*A*	*D*⋯*A*	*D*—H⋯*A*
O1—H1⋯N1^i^	0.82	2.10	2.830 (4)	149
O2—H2⋯N2	0.82	1.91	2.635 (4)	147

Symmetry code: (i) 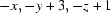.

**Fig. 1.**
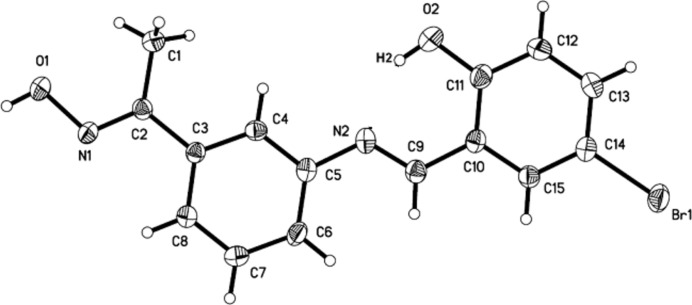
The molecule structure of the title compound with atom numbering. Displacement ellipsoids for non-hydrogen atoms are drawn at the 30% probability level.

**Fig. 2.**
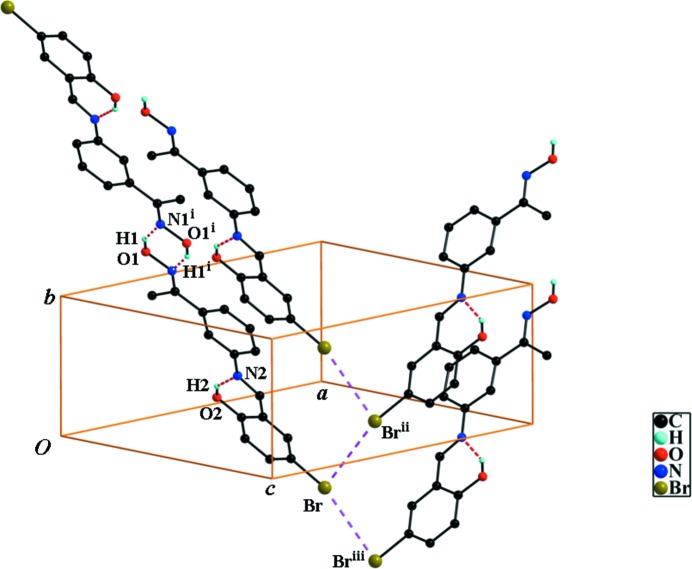
O—H..N and Br···Br interactions (dotted lines) in the crystal structure of the title compound. [Symmetry codes: (i) -x + 1, y + 1/2, -z + 3/2; (ii) -x + 1, y - 1/2, -z + 3/2; (iii) -x, -y + 3, -z + 1.]

## supplementary crystallographic information

### Comment

Schiff base ligands containing oxygen and imine nitrogen atoms have attracted much attention due to their variety of applications as well as their strong coordination capability (Dong *et al.*, 2007; Dong *et al.*, 2008; Wang *et al.*, 2009). The oxime compounds frequently exhibit versatility in organic, inorganic, bioinorganic, pigment, analytical, dyes and medical chemistry (Golovnia *et al.*, 2009; Liu *et al.*, 2008; Dong *et al.*, 2009*a*; Öztürk *et al.*, 2009). Owing to the importance of oxime-type compounds, we report the crystal structure of the title compound (Fig. 1).

In the crystal structure, all bond lengths and bond angles are in normal ranges. The molecule has a crystallographic inversion centre and the oxime unit adopts an E conformation with respect to the O—H group. The aniline ring (C3–C8) and phenol ring (C10–C15) are almost parallel each other, making a dihedral angle of 2.71 (1)°. The torsion angles of O1—N1—C2—C3 and C5—N2—C9—C10 are 178.5 (3) and -178.8 (3)°, respectively. In the crystal structure, a classical intramolecular O—H···N hydrogen bond forms a six-membered ring (Fig. 2 and Table 1). The crystal packing (Fig. 2) is stabilized by intermolecular O—H···N hydrogen bonds between the hydroxy groups and oxime N atoms, with a O1—H1···N1^i^ (Table 1). In addition, the crystal structure was further stabilized by weak intermolecular Br···Br^ii^ (Fig. 2) short interactions with a distance of 3.8768 (5) Å.

### Experimental

The title compound was synthesized according to an analogous method reported earlier (Dong *et al.*, 2009*b*). To an ethanol solution (5 ml) of 3-aminophenylethanone oxime (150.2 mg, 1.00 mmol) was added dropwise an ethanol solution (5 ml) of 5-bromosalicylaldehyde (201.1 mg, 1.00 mmol) then the yellow precipitate was obtained. The mixture solution was stirred at 328–333 K for 1 h. After cooling to room temperature, the precipitate was filtered off, dried *in vacuo* and purified by recrystallization from ethanol of solid. Yield: 54.01%, m. p. 459–461 K. Anal. Calc. for C_15_H_13_BrN_2_O_2_: C, 54.07; H, 3.93; N, 8.41. Found: C, 54.32; H, 4.01; N, 8.81.

Yellow needle-like single crystals suitable for X-ray diffraction studies were obtained by slow evaporation from a solution of dichloromethane at room temperature for about four weeks.

### Refinement

Non-H atoms were refined anisotropically. H atoms were treated as riding atoms with distances C—H = 0.96 Å (CH_3_), 0.93 Å (CH), O—H = 0.82 Å for (OH). The isotropic displacement parameters for all H atoms were set equal to 1.2 or 1.5 *U*_eq_ of the carrier atom.

### Crystal data

**Table:**

C_15_H_13_BrN_2_O_2_	*F*(000) = 672
*M**_r_* = 333.18	*D*_x_ = 1.549 Mg m^−^^3^
Monoclinic, *P*2_1_/*c*	Melting point = 459–461 K
Hall symbol: -p 2ybc	Mo *K*α radiation, λ = 0.71073 Å
*a* = 17.020 (2) Å	Cell parameters from 2173 reflections
*b* = 6.1676 (7) Å	θ = 2.7–23.8°
*c* = 13.693 (1) Å	µ = 2.88 mm^−^^1^
β = 96.461 (1)°	*T* = 298 K
*V* = 1428.3 (3) Å^3^	Needle-like, yellow
*Z* = 4	0.45 × 0.20 × 0.10 mm

### Data collection

**Table:**

Siemens SMART 1000 CCD area-detector diffractometer	2492 independent reflections
Radiation source: fine-focus sealed tube	1799 reflections with *I* > 2σ(*I*)
Detector resolution: 10.0 pixels mm^-1^	*R*_int_ = 0.051
φ and ω scans	θ_max_ = 25.0°, θ_min_ = 2.4°
Absorption correction: multi-scan (*SADABS*; Sheldrick, 1996)	*h* = −20→14
*T*_min_ = 0.357, *T*_max_ = 0.762	*k* = −7→7
6906 measured reflections	*l* = −15→16

### Refinement

**Table:**

Refinement on *F*^2^	Primary atom site location: structure-invariant direct methods
Least-squares matrix: full	Secondary atom site location: difference Fourier map
*R*[*F*^2^ > 2σ(*F*^2^)] = 0.039	Hydrogen site location: difference Fourier map
*wR*(*F*^2^) = 0.100	H-atom parameters constrained
*S* = 0.99	*w* = 1/[σ^2^(*F*_o_^2^) + (0.0498*P*)^2^] where *P* = (*F*_o_^2^ + 2*F*_c_^2^)/3
2492 reflections	(Δ/σ)_max_ < 0.001
182 parameters	Δρ_max_ = 0.47 e Å^−^^3^
0 restraints	Δρ_min_ = −0.32 e Å^−^^3^

### Special details

**Table:**

Geometry. All e.s.d.'s (except the e.s.d. in the dihedral angle between two l.s. planes) are estimated using the full covariance matrix. The cell e.s.d.'s are taken into account individually in the estimation of e.s.d.'s in distances, angles and torsion angles; correlations between e.s.d.'s in cell parameters are only used when they are defined by crystal symmetry. An approximate (isotropic) treatment of cell e.s.d.'s is used for estimating e.s.d.'s involving l.s. planes.
Refinement. Refinement of *F*^2^ against ALL reflections. The weighted *R*-factor *wR* and goodness of fit *S* are based on *F*^2^, conventional *R*-factors *R* are based on *F*, with *F* set to zero for negative *F*^2^. The threshold expression of *F*^2^ > σ(*F*^2^) is used only for calculating *R*-factors(gt) *etc*. and is not relevant to the choice of reflections for refinement. *R*-factors based on *F*^2^ are statistically about twice as large as those based on *F*, and *R*- factors based on ALL data will be even larger.

### Fractional atomic coordinates and isotropic or equivalent isotropic displacement parameters (Å^2^)

**Table:**

	*x*	*y*	*z*	*U*_iso_*/*U*_eq_	
Br	0.46909 (2)	−0.34895 (6)	0.66885 (3)	0.05662 (19)	
N1	0.04843 (18)	1.3049 (4)	0.46740 (19)	0.0451 (7)	
N2	0.25065 (15)	0.4933 (4)	0.52712 (19)	0.0416 (7)	
O1	0.01134 (16)	1.4311 (4)	0.38885 (16)	0.0609 (8)	
H1	−0.0096	1.5373	0.4110	0.091*	
O2	0.29339 (17)	0.2760 (5)	0.37605 (18)	0.0688 (8)	
H2	0.2718	0.3726	0.4044	0.103*	
C1	0.0818 (3)	1.0731 (7)	0.3317 (2)	0.0601 (11)	
H1A	0.0385	1.1454	0.2940	0.090*	
H1B	0.0753	0.9191	0.3247	0.090*	
H1C	0.1306	1.1160	0.3084	0.090*	
C2	0.0833 (2)	1.1340 (5)	0.4385 (2)	0.0355 (8)	
C3	0.12600 (18)	0.9934 (5)	0.5170 (2)	0.0328 (7)	
C4	0.16790 (19)	0.8077 (5)	0.4921 (2)	0.0361 (8)	
H4	0.1683	0.7723	0.4261	0.043*	
C5	0.2089 (2)	0.6749 (5)	0.5637 (2)	0.0372 (8)	
C6	0.2088 (2)	0.7269 (6)	0.6638 (2)	0.0457 (9)	
H6	0.2355	0.6402	0.7123	0.055*	
C7	0.1674 (2)	0.9129 (6)	0.6892 (2)	0.0541 (10)	
H7	0.1670	0.9486	0.7551	0.065*	
C8	0.1272 (2)	1.0439 (6)	0.6175 (2)	0.0439 (9)	
H8	0.1007	1.1664	0.6362	0.053*	
C9	0.2887 (2)	0.3533 (5)	0.5848 (2)	0.0402 (8)	
H9	0.2886	0.3675	0.6524	0.048*	
C10	0.33200 (19)	0.1729 (5)	0.5462 (2)	0.0370 (8)	
C11	0.3322 (2)	0.1382 (6)	0.4443 (3)	0.0455 (9)	
C12	0.3730 (2)	−0.0397 (6)	0.4104 (3)	0.0554 (10)	
H12	0.3726	−0.0625	0.3432	0.067*	
C13	0.4141 (2)	−0.1828 (6)	0.4770 (3)	0.0520 (10)	
H13	0.4413	−0.3000	0.4544	0.062*	
C14	0.4138 (2)	−0.1477 (5)	0.5778 (2)	0.0391 (8)	
C15	0.37336 (19)	0.0263 (5)	0.6123 (2)	0.0393 (8)	
H15	0.3735	0.0467	0.6797	0.047*	

### Atomic displacement parameters (Å^2^)

**Table:**

	*U*^11^	*U*^22^	*U*^33^	*U*^12^	*U*^13^	*U*^23^
Br	0.0571 (3)	0.0476 (3)	0.0643 (3)	0.01472 (19)	0.0030 (2)	0.00954 (19)
N1	0.056 (2)	0.0395 (17)	0.0372 (15)	0.0112 (15)	−0.0063 (14)	0.0022 (13)
N2	0.0357 (17)	0.0356 (17)	0.0537 (17)	0.0027 (13)	0.0058 (14)	0.0020 (13)
O1	0.083 (2)	0.0526 (16)	0.0436 (13)	0.0295 (15)	−0.0089 (13)	0.0039 (12)
O2	0.078 (2)	0.079 (2)	0.0479 (14)	0.0289 (17)	0.0001 (15)	0.0108 (14)
C1	0.081 (3)	0.062 (2)	0.0367 (19)	0.028 (2)	0.0017 (19)	−0.0001 (18)
C2	0.036 (2)	0.0329 (19)	0.0373 (17)	−0.0002 (15)	0.0027 (15)	−0.0020 (14)
C3	0.0309 (19)	0.0315 (18)	0.0353 (16)	0.0001 (14)	0.0005 (14)	0.0005 (14)
C4	0.037 (2)	0.037 (2)	0.0338 (16)	−0.0039 (15)	0.0021 (15)	−0.0024 (14)
C5	0.0311 (19)	0.0339 (19)	0.0462 (19)	−0.0012 (14)	0.0031 (15)	0.0022 (15)
C6	0.051 (2)	0.043 (2)	0.043 (2)	0.0113 (18)	0.0038 (17)	0.0141 (16)
C7	0.071 (3)	0.058 (2)	0.0331 (18)	0.014 (2)	0.0074 (18)	0.0027 (17)
C8	0.052 (2)	0.040 (2)	0.0395 (18)	0.0097 (17)	0.0060 (17)	0.0024 (16)
C9	0.040 (2)	0.0346 (19)	0.0455 (19)	−0.0010 (16)	0.0020 (16)	−0.0017 (16)
C10	0.0321 (19)	0.0332 (19)	0.0443 (18)	−0.0013 (15)	−0.0016 (15)	0.0004 (15)
C11	0.037 (2)	0.050 (2)	0.048 (2)	0.0033 (17)	0.0018 (17)	0.0052 (18)
C12	0.057 (3)	0.070 (3)	0.0408 (19)	0.012 (2)	0.0101 (18)	−0.0030 (19)
C13	0.051 (3)	0.051 (2)	0.055 (2)	0.0117 (18)	0.0109 (19)	−0.0053 (18)
C14	0.034 (2)	0.0331 (19)	0.050 (2)	−0.0024 (16)	0.0022 (16)	0.0017 (15)
C15	0.038 (2)	0.038 (2)	0.0411 (18)	0.0003 (16)	0.0013 (16)	−0.0041 (15)

### Geometric parameters (Å, °)

**Table:**

Br—C14	1.928 (3)	C4—H4	0.9300
Br—Br^i^	3.8768 (5)	C5—C6	1.409 (5)
Br—Br^ii^	3.8768 (5)	C6—C7	1.410 (5)
N1—C2	1.293 (4)	C6—H6	0.9300
N1—O1	1.417 (3)	C7—C8	1.390 (5)
N2—C9	1.294 (4)	C7—H7	0.9300
N2—C5	1.446 (4)	C8—H8	0.9300
O1—H1	0.8200	C9—C10	1.466 (4)
O2—C11	1.377 (4)	C9—H9	0.9300
O2—H2	0.8200	C10—C15	1.411 (4)
C1—C2	1.507 (4)	C10—C11	1.411 (5)
C1—H1A	0.9600	C11—C12	1.405 (5)
C1—H1B	0.9600	C12—C13	1.399 (5)
C1—H1C	0.9600	C12—H12	0.9300
C2—C3	1.504 (4)	C13—C14	1.398 (5)
C3—C8	1.409 (4)	C13—H13	0.9300
C3—C4	1.411 (4)	C14—C15	1.387 (4)
C4—C5	1.401 (4)	C15—H15	0.9300
			
C14—Br—Br^i^	86.56 (10)	C8—C7—C6	121.3 (3)
C14—Br—Br^ii^	164.58 (10)	C8—C7—H7	119.4
Br^i^—Br—Br^ii^	105.395 (19)	C6—C7—H7	119.4
C2—N1—O1	113.3 (3)	C7—C8—C3	120.9 (3)
C9—N2—C5	122.5 (3)	C7—C8—H8	119.6
N1—O1—H1	109.5	C3—C8—H8	119.6
C11—O2—H2	109.5	N2—C9—C10	121.6 (3)
C2—C1—H1A	109.5	N2—C9—H9	119.2
C2—C1—H1B	109.5	C10—C9—H9	119.2
H1A—C1—H1B	109.5	C15—C10—C11	118.7 (3)
C2—C1—H1C	109.5	C15—C10—C9	119.3 (3)
H1A—C1—H1C	109.5	C11—C10—C9	122.0 (3)
H1B—C1—H1C	109.5	O2—C11—C12	118.3 (3)
N1—C2—C3	116.9 (3)	O2—C11—C10	121.5 (3)
N1—C2—C1	122.8 (3)	C12—C11—C10	120.1 (3)
C3—C2—C1	120.2 (3)	C13—C12—C11	120.4 (3)
C8—C3—C4	117.6 (3)	C13—C12—H12	119.8
C8—C3—C2	121.6 (3)	C11—C12—H12	119.8
C4—C3—C2	120.8 (3)	C14—C13—C12	119.3 (3)
C5—C4—C3	122.0 (3)	C14—C13—H13	120.4
C5—C4—H4	119.0	C12—C13—H13	120.4
C3—C4—H4	119.0	C15—C14—C13	120.9 (3)
C4—C5—C6	119.5 (3)	C15—C14—Br	120.2 (2)
C4—C5—N2	115.8 (3)	C13—C14—Br	118.9 (2)
C6—C5—N2	124.6 (3)	C14—C15—C10	120.5 (3)
C5—C6—C7	118.7 (3)	C14—C15—H15	119.7
C5—C6—H6	120.6	C10—C15—H15	119.7
C7—C6—H6	120.6		
			
O1—N1—C2—C3	178.5 (3)	N2—C9—C10—C15	179.2 (3)
O1—N1—C2—C1	−1.9 (5)	N2—C9—C10—C11	−2.2 (5)
N1—C2—C3—C8	0.8 (5)	C15—C10—C11—O2	−179.6 (3)
C1—C2—C3—C8	−178.8 (3)	C9—C10—C11—O2	1.8 (5)
N1—C2—C3—C4	−177.9 (3)	C15—C10—C11—C12	0.1 (5)
C1—C2—C3—C4	2.5 (5)	C9—C10—C11—C12	−178.5 (3)
C8—C3—C4—C5	0.6 (5)	O2—C11—C12—C13	179.2 (4)
C2—C3—C4—C5	179.4 (3)	C10—C11—C12—C13	−0.5 (6)
C3—C4—C5—C6	−0.1 (5)	C11—C12—C13—C14	0.4 (6)
C3—C4—C5—N2	−178.6 (3)	C12—C13—C14—C15	0.0 (5)
C9—N2—C5—C4	−177.6 (3)	C12—C13—C14—Br	178.9 (3)
C9—N2—C5—C6	4.0 (5)	Br^i^—Br—C14—C15	−39.2 (3)
C4—C5—C6—C7	−0.3 (5)	Br^ii^—Br—C14—C15	102.3 (4)
N2—C5—C6—C7	178.1 (3)	Br^i^—Br—C14—C13	141.9 (3)
C5—C6—C7—C8	0.0 (6)	Br^ii^—Br—C14—C13	−76.7 (5)
C6—C7—C8—C3	0.6 (6)	C13—C14—C15—C10	−0.4 (5)
C4—C3—C8—C7	−0.9 (5)	Br—C14—C15—C10	−179.3 (2)
C2—C3—C8—C7	−179.6 (3)	C11—C10—C15—C14	0.3 (5)
C5—N2—C9—C10	−178.8 (3)	C9—C10—C15—C14	179.0 (3)

Symmetry codes: (i) −*x*+1, *y*+1/2, −*z*+3/2; (ii) −*x*+1, *y*−1/2, −*z*+3/2.

### Hydrogen-bond geometry (Å, °)

**Table:**

*D*—H···*A*	*D*—H	H···*A*	*D*···*A*	*D*—H···*A*
O1—H1···N1^iii^	0.82	2.10	2.830 (4)	149
O2—H2···N2	0.82	1.91	2.635 (4)	147

Symmetry codes: (iii) −*x*, −*y*+3, −*z*+1.
